# Neural Rhythms of Change: Long-Term Improvement after Successful Treatment in Children with Disruptive Behavior Problems

**DOI:** 10.1155/2015/873197

**Published:** 2015-07-16

**Authors:** Steven Woltering, Victoria Liao, Zhong-Xu Liu, Isabela Granic

**Affiliations:** ^1^Educational Psychology, Texas A&M University, College Station, TX 77843, USA; ^2^Applied Psychology and Human Development, University of Toronto, Toronto, ON, Canada M5S 2J7; ^3^Rotman Research Institute, The Baycrest Hospital, Toronto, ON, Canada M6A 2E1; ^4^Behavioural Science Institute, Radboud University, 6500 HE Nijmegen, Netherlands

## Abstract

Neural changes were investigated for children with disruptive behavior problems one year after a treatment program ended. Thirty-nine children and their parents visited the research lab before, after, and a year after treatment ended. During those lab visits, electroencephalography (EEG) was recorded during a challenging Go/No-go task. Treatment consisted of intensive 14-week combined cognitive behavioral therapy and parent management training sessions. For the analysis, participants were divided into long-term improvers (IMPs) and long-term nonimprovers (NIMPs) based on changes in their externalizing problem scores. The results showed early no-go theta power (4–8 Hz, 100–250 ms) decreased for long-term IMPs compared to NIMPs. When participants were divided based on changes in their comorbid internalizing symptoms, effects were stronger and reductions in theta power were found for early as well as later phases (250–650 ms). We provided preliminary evidence that theta power is a useful neural measure to trace behavioral change linked to improved self-regulation even up to a year after treatment ended. Results may have implications for the characterization of children with disruptive behavior problems and may lead to the development of novel markers of treatment success.

## 1. Introduction

The ultimate goal of treatment is to show benefits long after treatment ends. However, follow-up studies examining treatment outcomes for children with disruptive behavior problems (DBP) are rare and typically show small effect sizes [1, 2]. Furthermore, treatment studies often focus on testing the efficacy of a particular intervention by comparing an experimental treatment group with a control group (e.g., treatment as usual). However, this approach does not reveal the variability of outcomes* within* the treatment group itself: some children improve while others do not. Examining how improvers and nonimprovers differ in terms of the processes of change might be key in finding cost-saving predictors for effective treatment, the refinement of current treatment models, and the development of more reliable indices of long-term treatment success.

To explain what causes variability in long-term treatment outcomes, researchers have proposed political and socioeconomical as well as family and peer-relationship factors [3–5]. For example, Reyno and McGrath [6] showed that low family income was an important factor in predicting treatment outcomes for children with externalizing behavior problems. But the ability to change these factors is often small, especially for a child. What individuals* can* change, however, is how they cope with and manage to control the tonic stressors that surround them. This ability, to flexibly control one's own emotions and cognition in the service of (long-term) internal goals, is broadly defined as self-regulation [7]. Effective self-regulation skills are believed to function as buffers against stressors and build resilience in multiple domains. Studies have shown, for example, that effective self-regulation is associated with better academic performance and social functioning [8, 9]. Conversely, problems with self-regulation can manifest in severe internalizing or externalizing problem behaviors such as those related to DBP, anxiety, and attention disorders [7, 10, 11].

Increasing our measurement precision of self-regulation is important and the field has begun to look at neuroscientific techniques to provide the tools to measure and enrich our understanding of neural processes underlying self-regulation. The electroencephalography (EEG) technique is particularly sensitive in measuring the rapid cognitive processes that allow us to monitor and inhibit responses. For those reasons, event-related potentials (ERPs, averaged electrophysiological waveforms locked to an event), derived from the EEG, have traditionally been associated with self-regulatory processes during inhibition tasks. Differences in amplitudes of ERPs, for example, have been able to distinguish individuals with externalizing behavior problems from their typically developing peers [12–14].

Recently, researchers focus on event-related spectral perturbations (ERSPs; oscillatory patterns in the EEG waveform locked to events at the level of individual trials) to better understand human cognition. Such techniques capture rhythmic patterns derived from assemblies of neural populations which are widely considered intrinsic to brain function in general and crucial for the neural integration and processing of information in particular [15–17]. For example, alpha power (8–12 Hz) has been related to an active inhibition of task irrelevant brain areas during working memory tasks [18].

In the present paper our main objective was to focus on theta power. Theta power (4–8 Hz), derived from long-range, intercortical, or corticolimbic pathways, is understood as a binding rhythm that synchronizes multiple limbic and (neo)cortical brain regions [16, 19, 20]. In a review, Knyazev [16] concluded that theta power was mostly involved in memory and emotion regulation. In the field of working memory, for example, changes in theta power have been associated with the integration and maintenance of information [21, 22]. In the area of cognitive control, one recent study from our lab found that variations in later frontomidline theta power (after 250 ms) during the no-go trials of a Go/No-go task related to developmental changes in self-regulation [23]. Early frontomidline theta power, though prominent in the data, was not examined in this study. In another study, Lenartowicz et al. [24] compared children with ADHD and their typically developing peers using a working memory task and found differences in early frontomidline theta power during the high memory load.

We propose that, related to self-regulation, at an early stage of visual processing, theta power could act in recruiting attentional vigilance towards the encoding or monitoring of goal-relevant stimuli; and, at a later stage, theta power may be increasingly executive in nature and directly relate to dedicating attentional resources towards the inhibition of a response and subsequent evaluative processing (see also [25], for a recent review). Such an interpretation of early and later processing in the temporal dynamics of emotion regulation processing is in line with the recent ERP literature [14, 26] as well as a study exploring the chronometry of source activations localized to the medial prefrontal cortex [27]. In the latter study, an emotional Go/No-go task was used to track the chronometry of activation. Early window activity (around 200 ms) was interpreted as a bottom-up vigilance to perceived stimuli (i.e., manifested as a threat bias) whereas later activation was seen as more regulatory in nature.

Very little is known about the plasticity of the neural systems underlying self-regulation after successful treatment of DBP. To date, only two studies have been conducted that investigated neural changes using EEG with successful treatment of self-regulation. Lewis et al. [28] and Woltering et al. [13] used a Go/No-go task to examine children with DBP using ERP and source localization methods and found changes in neural activation after successful treatment. The treatment consisted of a combined cognitive behavioral therapy (CBT) and parent management training (PMT) program and used a broad range of evidence-based clinical techniques to train self-regulation skills (see [29], for overview). To the best of our knowledge, no treatment study has yet investigated long-term brain plasticity effects in children with DBP nor has any study examined changes in theta power after an intervention.

The present study built on two former studies from our lab that found neural changes in children who showed improvement on externalizing problem behaviors directly after treatment ended [13, 28]. In addition to examining improvements in externalizing behavior, we also aimed to investigate long-term changes in* internalizing* problems because emerging research indicates that anxiety may underlie the manifestation of DBP behaviors (see [30], for overview). Anxiety is also frequently comorbid with DBP, with up to 75% of children clinically referred for aggression exhibiting clinically elevated anxiety symptoms [30]. Although treatment was geared towards externalizing problems, therapeutic techniques that foster emotional control could also benefit underlying emotions of anxiety.

A number of families indicated that they were interested in being contacted a year after treatment ended, allowing us to investigate whether theta power would change for children who showed long-lasting improvement from their baseline session. Based on our previous studies [13, 28], which consistently found reductions in neural activation with successful treatment, directional hypotheses could be formulated. We hypothesized that long-term improvers would show a continuous reduction in frontomidline theta power from baseline to after treatment to follow-up compared to long-term nonimprovers. Consistent with a chronometry study using the same task, which found heightened neural activation for DBP children in early and later processing, we expected to find reductions in early and as well as later theta power [27]. Furthermore, we also hypothesized that long-term improvements in* internalizing* symptoms may be equally, or even more, sensitive to concomitant reductions in frontomidline theta activation.

## 2. Methods

### 2.1. Participants

Participants were recruited from two agencies that provided treatment for DBP. A total of 150 children between 8–12 years old and their families agreed to participate in the study. Sixty participants completed the follow-up assessment a year after treatment ended. Of these 60 participants, 39 (31 boys, 80%) had usable neural and behavioral data at pretreatment and follow-up sessions. These 39 participants constituted the sample for the present study. This select group did not differ from the initial sample at baseline on measures of age, sex, medication use, demographics, or on severity of problem behavior as determined by *t*-tests. See Table 1 for a more detailed description of the sample, including socioeconomic and ethnic demographics. The vast majority of medication use consisted of stimulant medication (either Concerta or Ritalin).

Participants were referred to treatment agencies by mental health professionals, teachers, police, and/or parents. Inclusion criteria consisted of scores on or above the borderline-clinical range (84th percentile) on the externalizing scale of the child behavior checklist (CBCL; [31]). Exclusion criteria consisted of significant cognitive impairment, such as a persistent developmental delay (no participants were excluded on these grounds). The study was approved by the research Ethics Board of the University of Toronto.

### 2.2. Intervention

An evidence-based treatment program called SNAP (Stop Now and Plan; Augimeri, Walsh, Levene, Sewell, and Rajca, 2014) was delivered to children (CBT) and parents (PMT). The SNAP program has undergone evaluations demonstrating positive treatment outcomes evidenced at least to 6- and 12-month follow-up periods [32, 33]. Three-hour-long weekly group therapy sessions were held separately for the children and their parents at the community agencies for 14 weeks. CBT targeted effective regulation of emotional and other behaviors through well-documented strategies such as cognitive restructuring, problem solving, role-playing, and social and token reinforcements, as well as generalization activities [34, 35]. PMT promoted positive parenting practices such as skill encouragement, problem solving, and monitoring, as well as the replacement of coercive or lax discipline strategies with mild sanctions targeting misbehavior [36–38]. Therapists were either social workers, child-care workers, and M.A.- or Ph.D.-level clinical psychology students. Therapists were trained in the PMT and CBT protocols and regularly supervised to ensure compliance with the treatment model.

### 2.3. Procedure

Behavioral and EEG data were collected 2 weeks both before and after the 14-week treatment sessions. Follow-up sessions occurred 12 months after treatment had ended. During lab sessions, children were accompanied to the lab by a parent. After a brief introduction to the testing environment, parental consent and child assent were obtained (first session only) in accordance with the guidelines of the 1964 Declaration of Helsinki (World Medical Organization). Parents were seated in an adjacent room and asked to complete the CBCL. For the first two lab sessions, a procedure followed which involved a discussion with the parent and a battery of executive function tasks. The results of these data are not used for this report (see [39, 40], for more details). Next, children were informed that they could win a big prize for playing the EEG “computer game” and were shown two toy bins. One of the bins contained small, undesirable toys. A second, “big prize” bin contained a wide selection of more desirable, age-appropriate toys such as action figures, stuffed animals, games, and $10 gift certificates from a local music/computer game store. As part of a mood induction, the children were informed that successful performance (accumulation of points) in the game would allow them to pick a prize from the big prize bin but poor performance would limit their choice to the less desirable bin.

In the EEG recording room, children were seated in front of a computer screen. The height of the seat and the angle of the chin rest were adjusted to align the children's eyes to the center of the computer screen after which children were instructed on the Go/No-go task. At the end of the session, all participants received the big prize, regardless of performance.

### 2.4. Measures

*Questionnaire Measure*. The CBCL [31] is a highly reliable and commonly used parent report of child problem behavior yielding standardized *T*-scores for scales such as internalizing and externalizing behavior problems. Borderline-clinical scores for these subscales constitute *T*-scores ranging from 60 to 63 (starting from 84th percentile) and anything above 63 is considered clinical (98th percentile).

*Go/No-go Task*. An adapted version of a previously developed Go/No-go task was used for the present study [41]. The task was presented using E-Prime software (Psychological Software Tools, Pittsburgh, PA). Participants were required to press a button as fast as possible whenever a letter appeared on the screen (the go condition) and withhold responding whenever a letter was repeated a second time in succession (the no-go condition). Children used the index finger of their dominant hand. In order to provide the same level of challenge for all participants at all ages and to obtain a sufficient number of correct no-go trials for our analyses, a dynamic adjustment of the stimulus time was used in the task. The no-go error-rate for the task was maintained at 50%  ±  10% by dynamically adjusting the stimulus duration. For example, stimulus duration was increased with each erroneous response made on no-go trials and decreased following correct no-go trials, but only when the no-go trial followed a correct go trial. For each block, accumulated points were displayed approximately every 20 trials in the center of the computer screen. Points were added for correct no-go responses and deducted for response errors on both go and no-go trials. Error feedback was provided by a red bar in the middle of the screen for 200 ms following incorrect responses, omitted responses, and late responses. To minimize intertrial interference clear time was introduced at the end of each trial during which no stimulus was presented (500 ms after response; 400 ms after no response).

Children were presented with a practice block followed by three blocks of trials in a fixed order (blocks A, B, and C). In blocks A and C children gained points quite steadily. These blocks were structurally identical, each consisting of 200 trials, including 66 no-go trials (2 : 1 ratio of go to no-go), in pseudorandom sequence. In block B, children immediately began losing all (or almost all) points due to a change in the point-adjustment algorithm as well as a reduction in overall stimulus duration (we point out that this has not always been clear in previous publications from our lab; this prevents direct comparisons between block B and other blocks on performance). The loss of points was intended to induce negative emotion, such as anxiety and/or frustration. To limit the intensity and duration of children's distress, block B consisted of only 150 trials, including 40 no-go trials. Children were reminded at the beginning of the task and at the onset of each block that a high number of points were required to win the “big prize.” At the end of the task, all children were told that they would receive the big prize. At the end of the last and third session, a debriefing procedure explained to the child that the second block was rigged and that it was impossible to accumulate points. Block B was not analyzed for the present study due to low trial counts (the mean no-go trial count was 9) and because it was structurally different from the other blocks.

### 2.5. EEG Analysis

EEG data were collected using a 129-channel sensor net (GSN 200; Electrical Geodesics Inc., Eugene, OR). The sampling rate of the data digitization was 250 Hz and impedance values had to be below 50 KΩ before recording could begin [42]. All channels were referenced to C_z_ during recording. Then, data were filtered to the frequency range of 1–30 Hz off-line, using an FIR band-pass filter. The filtered data were then segmented into trial segments from 400 ms before to 1000 ms after the stimulus onset.

Data cleaning followed a standard procedure where an automated algorithm was run using an EGI Netstation artifacting tool (Electrical Geodesics, Inc., Eugene, OR). Channels were automatically marked bad when they exceeded a transition threshold of 200 *μ*v over the entire segment (max–min). After running a 20 ms moving-average smoothing algorithm, remaining eye blinks were detected when the vertical eye channels exceeded a threshold of 150 *μ*v (max–min) within a 160 ms (moving) time window within each trial. Eye movements were detected when horizontal eye channels exceeded a threshold of 100 *μ*v (max–min) over a 200 ms time window (HEOG and VEOG channels were recorded simultaneously with the EEG). Furthermore, each segment of the EEG was excluded from averaging if 15 or more channels were rejected. If a channel was marked bad in 25% of the trials, it was considered suspicious and marked bad for all trials. Trained research assistants, blind to the hypotheses, checked each file for accuracy.

Next, bad channels were interpolated using spherical splines on a trial by trial basis. Data were subsequently average-referenced (this method most closely approaches a reference-free montage using a dense array system; [43]) and exported to MATLAB (The Mathworks, Inc.). ERP averaging and baseline correction was also conducted for the P3 component (mean of 500–800 ms, for the midline electrodes: 6, 11, and C_z_).

To calculate theta power, time-frequency decomposition was performed on individual no-go trial data using short-time fast Fourier transformation (FFT) with a moving Hanning window (EEGLAB, [44]). Through a logarithmic transformation, power values were in decibel (dB) units. The output frequency ranged from 1.95 Hz to 29.3 Hz divided into 15 linear-spaced frequency bands with 1.95 Hz steps. Theta power was centered around 3.9, 5.8, and 7.8 Hz. In the time domain, the FFT output was calculated for 280 time points which covered a range of 272 ms before to 872 ms after the stimulus. Baseline correction was done by subtracting prestimulus theta power (defined at −150–0 ms) from the poststimulus period (see also [23]).

To prevent unbalanced trial counts across sessions from influencing the results for within-subjects analyses, all trial counts were set to the lowest value across all sessions (a random selection was made when trials had to be reduced for certain time points in order to make them equal across all time points). There was no difference in trial counts between IMPs (m = 22.5, sd = 8.2) and NIMPs (m = 22.3, sd = 7.2). No subjects had fewer than 10 trials.

**Figure 1:**
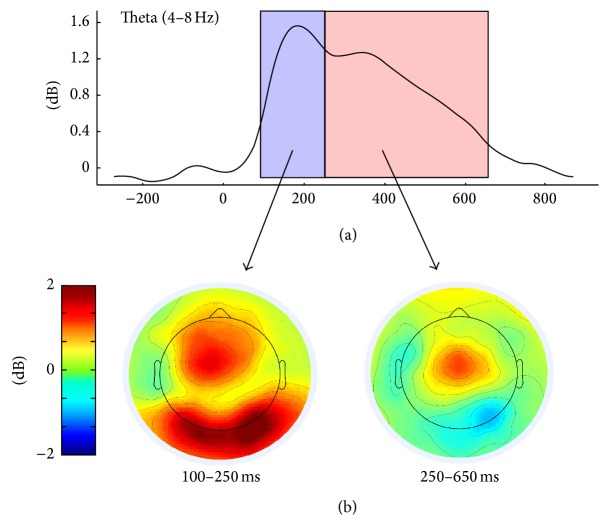
(a) Plot showing the grand average of theta power (in dB) across time. (b) The topo plots for theta power are shown for the early (left) and later peak (right).

A grand average (GA) waveform of theta power in the 4–8 Hz range was produced to aid in determining the region and time windows of interest before any of the improver status analyses were explored. Based on previous research (see [13]) and the GA-plot, frontomidline electrodes C_z_, FC_z_, and F_z_ were chosen as the sites of interest. For the time windows of interest, two peaks were distinguished from the GA-plot: an early one, ranging from 100 to 250 ms, and a later one, ranging from 250 to 650 ms. As shown in Figure 1, the early peak (100–250 ms) of frontomidline activation also features theta activation in posterior sites suggestive of occipitoparietal activation. We will interpret variation in theta power during this early period as differences in attentional resources being dedicated to binding percepts (e.g., the stimuli) with motivational states. This may be reflected in behavior by a vigilance of attention in the monitoring of the task. Although not initially planned, based on Figure 1, we will also conduct additional analyses investigating theta power at posterior sites during the earlier time window (theta power was most consistently centered around O_1_, O_z_, and O_2_). The correlation between theta power in frontal and posterior sites was *r*(39) = .82, *p* < .001, suggesting communication between these regions. The later theta power peak (250–650 ms) will be interpreted as more executive in nature and can reflect attentional resources being dedicated to inhibiting the response as well as an evaluation of outcomes (see also rationale in introduction).

### 2.6. Group Classification

To classify our sample into long-term improvers (IMPs) and nonimprovers (NIMPs), we performed a median split on the change-scores from baseline to follow-up on the externalizing subscale of the CBCL. Considering our low sample size, this method ensured that we had a sizable amount of IMPs and NIMPs to conduct our planned analyses. A similar method was used to classify IMPs and NIMPs based on long-term changes in internalizing symptoms. Seventy-two percent of all participants were consistently classified as IMPs or NIMPs regardless of whether the grouping was performed by externalizing or internalizing symptom improvement. No statistically significant differences were found between the IMPs and the NIMPs in age, sex, medication, and demographic variables related to ethnicity, parental education, and family income (see also Table 1).

### 2.7. Statistical Analysis

Outlier analyses were performed on each of the variables whereby data points were removed from further analysis if they were more than three standard deviations from the mean. No outliers were present for the variables in the present study. Block was explored as a factor in all main neural analyses but did not appear to be significant in any of our analyses. Therefore, to reduce degrees of freedom, we have combined the data for blocks A and C. Greenhouse-Geisser corrected statistics were reported when assumptions of sphericity were violated for the Repeated Measures ANOVA. Partial eta-squared values (*η* ^2^) were computed to ascertain effect size. According to [45], partial *η* ^2^ = .01 corresponds to a small effect, partial *η* ^2^ = .10 corresponds to a medium effect, and partial *η* ^2^ = .25 represents a large effect.

## 3. Results

### 3.1. Questionnaire and Behavioral Performance

Externalizing symptoms generally decreased for the whole sample across the three sessions as shown by a one-way within-subjects ANOVA, *F*(2,34) = 21.60, *p* < .001, partial *η* ^2^ = .56. A similar pattern could be seen for internalizing symptomatology, *F*(2,34) = 7.82, *p* = .002, partial *η* ^2^. We note that although our sample was selected for externalizing symptomatology, the mean *T*-score for internalizing symptomatology was well in the borderline-clinical range and almost reached standard clinical levels of impairment (m = 63.9, sd = 6.9).  Table 2 shows the CBCL *T*-scores broken down for long-term improver status group for each of the sessions. Differences between long-term improver status were tested at each session using *t*-tests.

To investigate changes in the behavioral performance measures during the Go/No-go task, mixed model Repeated Measures ANOVAs were run with session (3 levels: pretreatment, posttreatment, and follow-up) and group (2 levels: IMPs and NIMPs) as within- and between-subject factors, respectively. Whether groups were divided by changes in their externalizing or internalizing scores, no statistically significant interaction effects were found between IMPs and NIMPs. In these analyses, main effects of session were found for go and no-go accuracy as well as for reaction time, showing that accuracy increased and reaction times became faster (all *p*'s < .01).  Table 2 shows the behavioral performance data at each time point with differences of long-term improver status tested using *t*-tests.

### 3.2. Theta Power

#### 3.2.1. Grouping by Externalizing Problems

**Figure 2:**
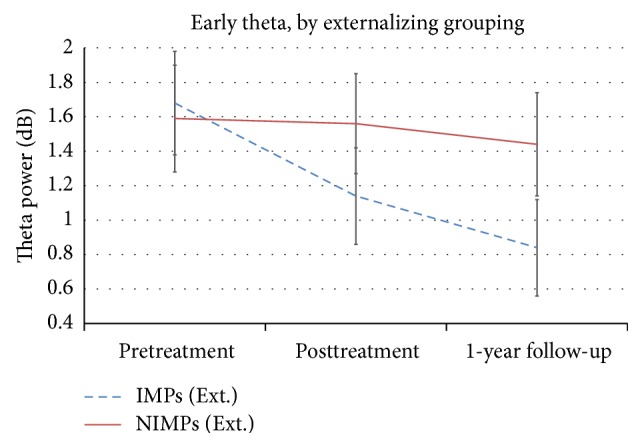
Line plot showing early theta power (in dB) for the externalizing (Ext.) grouping across all session time points broken down for improver status. NIMPs: nonimprovers; IMPs: improvers.

To investigate whether IMPs, grouped by their changes in externalizing problems, were showing differences in frontomidline theta power across time compared to NIMPs, mixed model Repeated Measures ANOVAs were run with session (3 levels: pretreatment, posttreatment, and follow-up) and group (2 levels: IMPs and NIMPs) as factors. For the first theta power peak, a main effect of session was found, *F*(2,33) = 3.72, *p* = .035, partial *η* ^2^ = .18, showing a general decrease in theta power. There was no statistically significant group-by-session interaction effect (*p* = .17), but because we hypothesized a decrease in IMPs compared to NIMPs, we were justified in investigating the planned contrasts. As predicted, IMPs showed a significant decrease in theta power from pretreatment to follow-up (*p* = .002) whereas this was not the case for NIMPs (*p* = .56). Moreover, a marginally significant effect was also found for IMPs from pretreatment to posttreatment (*p* = .095).  Figure 2 shows the theta power across sessions for IMPs and NIMPs for the first peak.

As an additional analysis, posterior theta power was also investigated. Though patterns in the data were similar, there was no statistically significant effect group-by-session interaction effect.

**Figure 3:**
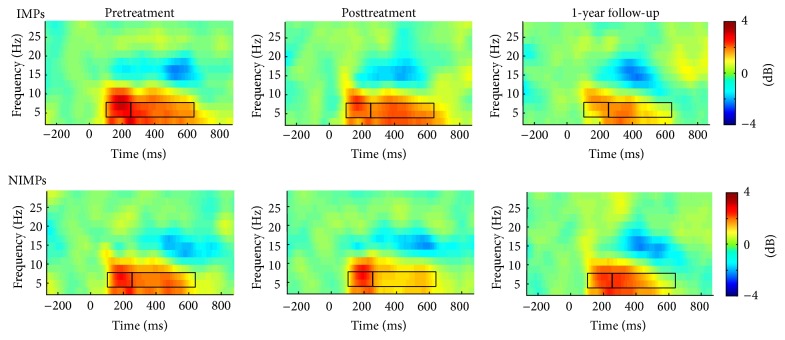
Time-frequency plots showing theta power (in dB) at each session (pretreatment, posttreatment, and follow-up) for improvers (IMPs) and nonimprovers (NIMPs) for our analysis classifying improvement status by externalizing changes. Boxes delineate the early and later peak of the theta band.

No statistically significant main or interaction effects were found for the second theta peak.  Figure 3 shows the time-frequency plots across all time points for session and group.

#### 3.2.2. Grouping by Internalizing Problems

When children were grouped by their level of change in theta power based on internalizing problems a main effect of session was found for the first peak, *F*(2,33) = 3.87, *p* = .031, partial *η* ^2^ = .19, showing a decrease in theta power across sessions. A session-by-group interaction effect was found at the level of a trend, *F*(2,33) = 3.00, *p* = .064, partial *η* ^2^ = .19, whereby planned contrasts revealed a statistically significant decrease from pretreatment to follow-up (*p* = .001) for IMPs only (NIMPs, *p* = .81). No statistically significant effects were found for posterior theta.

**Figure 4:**
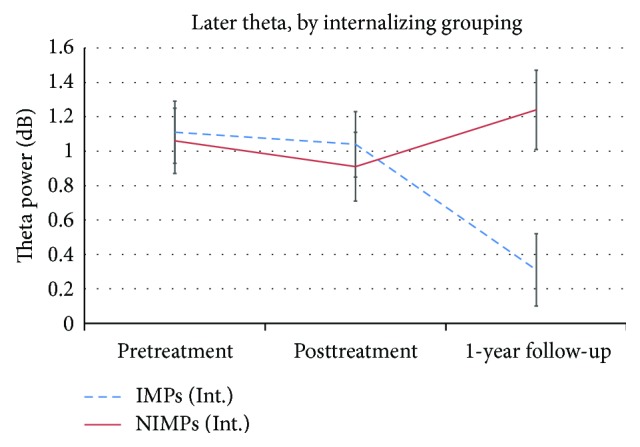
Line plot showing later theta power (in dB) for the internalizing (Int.) grouping across all session time points broken down for improver status. NIMPs: nonimprovers; IMPs: improvers.

For the second peak, a statistically significant session-by-group interaction effect was found, *F*(2,33) = 4.57, *p* = .018, partial *η* ^2^ = .19, showing decreases in theta power for IMPs from pretreatment to follow-up (*p* = .001) and from posttreatment to follow-up (*p* = .01).  Figure 4 plots the theta power across sessions for IMPs and NIMPs for the second peak.

**Figure 5:**
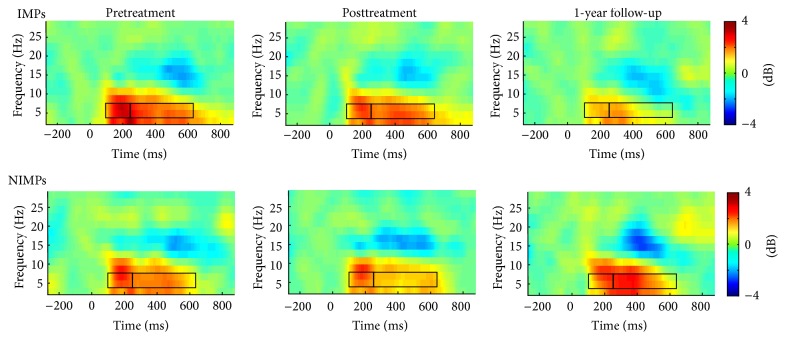
Time-frequency plots showing theta power (in dB) at each session (pretreatment, posttreatment, and follow-up) for improvers (IMPs) and nonimprovers (NIMPs) for our main analysis classifying improvement status by internalizing changes. Boxes delineate the early and later peak of the theta band.

Figure 5 shows the time-frequency plots across all time points for each session and group when participants were grouped by changes in internalizing scores. Correlations were also run to test whether the changes in externalizing or internalizing scores were directly associated with changes in early and later theta power. As expected, marginally significant decreases in later theta power were associated with reductions in internalizing symptoms, *r*(39) = .28, *p* = .08. Remaining correlations did not reach standard levels of statistical significance.

To show that results of theta were not solely due to relatively slow ERP components such as the P3, the main analyses were repeated with the P3 ERP component. No significant interaction effects were found for the externalizing or internalizing grouping analyses, suggesting that theta power adds unique variance over and above the ERP.

## 4. Discussion

We set out to test whether long-term behavioral improvement would be reflected in changes in neural theta power in children with DBP who participated in a treatment aimed at improving self-regulation. Our findings show reductions in neural activation only for those children who showed long-term improvement in their externalizing symptoms. These findings are in line with previous studies from our lab investigating neural changes* directly after* treatment [13, 28] and suggest increased neural efficiency when children improve. That these effects occurred during early theta power may suggest that the brains of long-term improvers have a more efficient communication between occipitoparietal regions involved in perception and the frontolimbic systems mediating motivation and monitoring. And that these effects occurred in the absence of behavioral performance differences suggests increased neural efficiency as a similar performance is achieved with an equal amount of neural resources. These findings add to the extant literature as previous studies, using ERP, have typically found changes at a later stage of processing [13].

When participants were grouped on the basis of long-term improvement in their internalizing symptomatology, a similar but stronger pattern was found for long-term improvers. Next to improvers showing decreased theta power activation during the early phase of processing, we also found decreases during later theta, suggesting increased neural efficiency in more executive processing related to inhibition and evaluation. The finding that theta power was more sensitive to changes in internalizing than externalizing symptoms suggests that theta power may be more strongly connected to underlying effects of anxiety. We think that this latter finding has consequences for the characterization of our sample as well as the explanation of our effects.

We interpret our neural effects in long-term IMPs to mean an increased efficiency in neural systems mediating a hypervigilant and overcontrolling style of self-regulation. There is a growing consensus that comorbid anxiety in children with DBP is not just an auxiliary phenomenon but that it may drive and maintain externalizing problems (see [30], for overview). Anxiety problems are characterized by a continuous hypervigilance to threat, excessive worry, and an overcontrolled style of self-regulation [11, 46]. The externalizing problem behaviors in our sample could be explained through a depleted self-regulatory capacity due to prolonged anxiety which could result in aggressive outbursts (e.g., consistent with Baumeister's ego-depletion model, [47]). At a neural level, the heightened theta power may reflect overactive frontolimbic circuits interacting at an early stage with perceptual processes which could underlie the threat-focused attentional biases [48, 49] and at a later stage with executive processes underlying the inefficient inhibitory control and evaluative processes common in anxiety [11, 50]. The reduction of theta power after successful treatment could reflect normalization in the overactive frontolimbic systems that underlie these hypervigilant states and the overcontrolled style of self-regulation. We suggest, at a behavioral level, that this would relieve some of the tension and rigidity these children bring to social situations and, according to the anxiety hypothesis of aggression [30, 51], lead to fewer bouts of aggression and other externalizing problem behaviors.

## 5. Implications

These results, particularly if they are replicated by independent groups, have implications for the characterization and treatment of children with DBP. The finding that internalizing symptoms, as opposed to externalizing symptoms, seemed more sensitive to long-lasting neural changes reinforces the notion that anxiety may lie at the root of DBP. This may suggest that DBP treatment should focus on targeting anxiety as that may more effectively relieve children of the underlying problems they experience. We are also a step closer to finding reliable neural markers of treatment efficacy. Such measures directly tapping into the neural systems underlying the cognitive processes of self-regulation could, in the future, complement traditional indices of treatment success. We suggest a multimethod approach, where a set of indices based on ERP [13, 14], source localization [28, 52], psychophysiology [39, 40], and power [23] may together form a powerful toolkit for predicting and understanding treatment success.

## 6. Limitations and Considerations

A number of considerations apply to this study. First, the current study was not intended nor conducted as a randomized controlled trial (RCT). Our primary interest was to look at individual differences in responses to treatment rather than in assessing the impact of the model of treatment delivery on outcomes. The clinical population thus functioned as their own “control.” This meant, however, that caution must be taken when drawing conclusions about treatment efficacy and statements about what* caused* these children to show improvement in the present study. We would also like to point out that it was not possible, due to the low sample size, to get reliable data on maintenance effects, that is, taking only the improvers at post and investigating only* their* progress a year later. A future study, using a much larger sample size and an RCT design, would be able to more definitively attribute changes to treatment* and* provide finer delineation, at neural and behavioral level, of the treatment effects across time.

Second, a limitation of the study is the lack of formal clinical diagnoses in accordance with DSM criteria for conduct disorder, oppositional defiant disorder, ADHD, and/or mood disorders. This was a limitation that was hard to remedy for practical reasons. To ensure that our results were relevant to “real world” practice, the study was conducted in partnership with community-based child and family agencies that implement evidence-based interventions. As a result, the study was constrained to the protocols that these agencies already had in place. Children referred for treatment to these agencies did not regularly undergo full psychiatric assessments. Instead, a number of standardized measures were used to assess children's and parents' clinically relevant symptoms and functioning.

Third, we did not have complete verifiable information on treatment programs that participants may have signed up for in the year between our second and third lab sessions. This information could have helped interpret what made treatment successful. Regardless, the strength of the current design is geared towards investigating what changes with successful compared to unsuccessful outcomes and is less suitable for determining the causal reasons for that change.

Last, although we have focused on theta power, we acknowledge that other oscillations could also play a role in the neural plasticity of self-regulatory processes. In fact, researchers have also suggested these different oscillations are not independent but interact with each other in reciprocal ways [53, 54]. We believe such approaches, incorporating multiple bands and their interactions, are a fruitful approach for future studies.

## 7. Conclusion

To the best of our knowledge, this is the only intervention study to date investigating neural correlates of self-regulation in children with DBP a year after treatment ended. The study provides preliminary support for the idea that long-term changes can be found in frontomidline theta power activation patterns mediating behavior underlying self-regulation. The results may help provide more comprehensive understanding of children with DBP and work towards neural indices that aid with the diagnosis and efficacy of treatment.

## Figures and Tables

**Table 1:** Age, sex, medication, and demographics information broken down for IMP and NIMPs across groupings based on externalizing and internalizing symptomatology.

	Externalizers (*n* = 39)		Internalizers (*n* = 39)	
	Nonimprovers (*n* = 18)	Improvers (*n* = 21)	Nonimprovers (*n* = 17)	Improvers (*n* = 22)
Age (years)	9.39 (1.04)	9.67 (1.28)	9.47 (.95)	9.59 (1.33)
Sex (% males)	83.3%	76.2%	82.4%	77.3%
Medication				
Stimulant	6 (33.3%)	4 (19.0%)	6 (33.3%)	4 (18.2%)
Other	3 (16.7%)	3 (14.3%)	2 (11.8%)	3 (13.6%)
None	9 (50.0%)	14 (66.7%)	9 (52.9%)	14 (63.6%)
Ethnicity				
European	13 (72.2%)	18 (85.7%)	14 (82.4%)	17 (77.3%)
African/Caribbean	1 (5.6%)	2 (9.5%)	—	3 (13.6%)
Other	4 (22.2%)	1 (4.8%)	3 (17.6%)	2 (9.1%)
Mother's education				
High school or less	5 (27.8%)	8 (38.1%)	5 (29.4%)	8 (36.4%)
Community college	10 (55.6%)	7 (33.3%)	7 (41.2%)	10 (45.5%)
University or above	3 (16.7%)	5 (23.8%)	5 (29.4%)	3 (13.6%)
Other/unknown	—	1 (4.8%)	—	1 (4.5%)
Father's education				
High school or less	5 (35.7%)	10 (58.8%)	7 (53.8%)	8 (44.4%)
Community college	7 (50.0%)	2 (11.8%)	3 (23.1%)	6 (33.3%)
University or above	1 (7.1%)	4 (23.5%)	2 (15.4%)	3 (16.7%)
Other/unknown	1 (7.1%)	1 (5.9%)	1 (7.7%)	1 (5.6%)
Family income ($)				
0–29,999	6 (33.3%)	4 (23.5%)	5 (35.7%)	5 (23.8%)
30,000–59,999	1 (5.6%)	5 (29.4%)	2 (14.3%)	4 (19.0%)
60,000 or above	11 (61.1%)	8 (47.1%)	7 (50.0%)	12 (57.1%)

**Table 2:** CBCL and behavioral performance data (mean and standard deviations) during the Go/No-go task before treatment, after treatment, and for follow-up, broken down for when groups were divided on changes in externalizing or internalizing symptoms. NIMPs: nonimprovers; IMPs: improvers. Statistical differences between long-term improvers (IMPs) and nonimprovers (NIMPs) were tested at each time point using *t*-tests.

	Before (*n* = 39)				After (*n* = 36)				Follow-up (*n* = 39)			
	Externalizers		Internalizers		Externalizers		Internalizers		Externalizers		Internalizers	
	NIMPs	IMPs	NIMPs	IMPs	NIMPs	IMPs	NIMPs	IMPs	NIMPs	IMPs	NIMPs	IMPs
CBCL externalizing	72.6 (3.80)	71.62 (4.91)	72.24 (4.91)	72.00 (4.11)	70.65 (5.38)^*∗*^	65.26 (7.71)^*∗*^	69.65 (6.67)	66.16 (7.36)	71.39 (5.22)^*∗*^	58.76 (6.29)^*∗*^	67.82 (7.87)^*∗*^	62.09 (8.43)^*∗*^

CBCL internalizing	64.67 (6.37)	63.52 (7.16)	63.82 (5.62)	64.23 (7.63)	62.76 (10.30)	59.00 (10.23)	65.06 (8.63)^*∗*^	56.95 (10.54)^*∗*^	62.33 (9.66)^*∗*^	54.00 (9.77)^*∗*^	65.06 (6.53)^*∗*^	52.57 (9.56)^*∗*^

Go accuracy	.82 (.10)	.87 (.07)	.85 (.11)	.84 (.07)	.87 (.94)	.87 (.63)	.89 (.07)	.85 (.08)	.89 (.07)	.90 (.06)	.92 (.05)^*∗*^	.87 (.07)^*∗*^

no-go accuracy	.53 (.12)	.52 (.08)	.50 (.13)	.54 (.07)	.57 (.12)	.59 (.09)	.56 (.10)	.61 (.10)	.60 (.11)	.61 (.10)	.56 (.11)^*∗*^	.63 (.09)^*∗*^

Reaction time (Go)	417 (79)^*∗*^	410 (49)^*∗*^	412 (78)	415 (52)	371 (70)^*∗*^	360 (43)^*∗*^	364 (70)	366 (44)	338 (44)	335 (38)	337 (47)	336 (35)

^*∗*^ *p* < .05.
